# Feline vector-borne pathogens in Iran

**DOI:** 10.1186/s13071-025-06788-x

**Published:** 2025-04-28

**Authors:** Alireza Sazmand, Mariaelisa Carbonara, Leili Moradi, Pardis Almasi, Shiva Saruei, Mozhdeh Moradi-Jam, Anahita Akhondi, Parmida Malekzadeh, Soheila Ghaharzade-Mahabadi, Amin Bakhshani, Omid Chashnigir, Baharak Akhtardanesh, Hamidreza Moosavian, Mohammad Ramezani, Darioush Shirani, Livia Perles, Jairo Alfonso Mendoza-Roldan, Zainab Sadeghi-Dehkordi, Reza Nabavi, Fateme Jalousian, Domenico Otranto

**Affiliations:** 1 Department of Pathobiology, Faculty of Veterinary Medicine, Bu-Ali Sina University, Hamedan, 6517658978 Iran; 2 Department of Veterinary Medicine, University of Bari, Valenzano, 70010 Bari, Italy; 3 Department of Parasitology, Faculty of Veterinary Medicine, University of Tehran, Tehran, Iran; 4 Department of Pathobiology, Faculty of Veterinary Medicine, Ferdowsi University of Mashhad, Mashhad, Iran; 5 Dr. Chashnigir Veterinary Clinic, Yazd, Iran; 6 Department of Clinical Sciences, Faculty of Veterinary Medicine, Shahid Bahonar University, Kerman, Iran; 7 Department of Clinical Pathology, Faculty of Veterinary Medicine, University of Tehran, Tehran, Iran; 8 Department of Pathobiology, Faculty of Veterinary Medicine, Lorestan University, Khorramabad, Iran; 9 Department of Internal Medicine, Faculty of Veterinary Medicine, University of Tehran, Tehran, Iran; 10 The Iranian Museum of Parasitology, Faculty of Veterinary Medicine, University of Tehran, Tehran, Iran; 11 Department of Veterinary Clinical Sciences, City University of Hong Kong, Kowloon Tong, Hong Kong, China

**Keywords:** Domestic felids, Vector-borne pathogens, *Dirofilaria* spp., *Hepatozoon* spp., *Leishmania* spp., One health

## Abstract

**Background:**

Feline vector-borne pathogens (FeVBPs) are common in tropical and subtropical countries, mainly due to favorable climate conditions for arthropod perpetuation coupled with limited preventive measures. However, data regarding the actual burden of these infections among cats are still scarce compared with dogs. The present study aimed to provide an overview of the prevalence of FeVBPs infections in Iran.

**Methods:**

From December 2018 to February 2023, a total of 848 cats of both sexes, different ages, and with outdoor lifestyle living in 7 provinces of Iran were blood sampled and molecularly screened for *Hepatozoon* spp., *Babesia* spp., *Cytauxzoon* spp., *Dirofilaria* spp., and *Leishmania* spp.

**Results:**

Overall, 5.4% of cats scored positive for at least one VBP, with *Hepatozoon* spp. being the most common (3.8%), followed by *Leishmania* spp. (2.5%) and *Dirofilaria immitis* (0.7%). The *Hepatozoon*-positive cats lived in localities from the eastern, western, and central-northern regions; most of them (*n* = 25) were infected by *Hepatozoon felis,* and the remaining (*n* = 3) by *Hepatozoon canis*. *Leishmania* spp.-infected cats were detected from the east, center, and west of the country, while *D. immitis*-positive animals lived in central-north areas.

**Conclusions:**

To our knowledge, this is the first large-scale molecular epidemiology study of vector-borne pathogens in cats in Iran. The circulation of several VBPs, including those with zoonotic potential (i.e., *D*. *immitis* and *Leishmania* spp.) highlights the importance of endo- and ectoparasite control measures in owned cats and suggests that controlling the population of feral animals (e.g., through spaying and neutering campaigns) would contribute to reducing the risk of transmission of VBPs.

**Graphical abstract:**

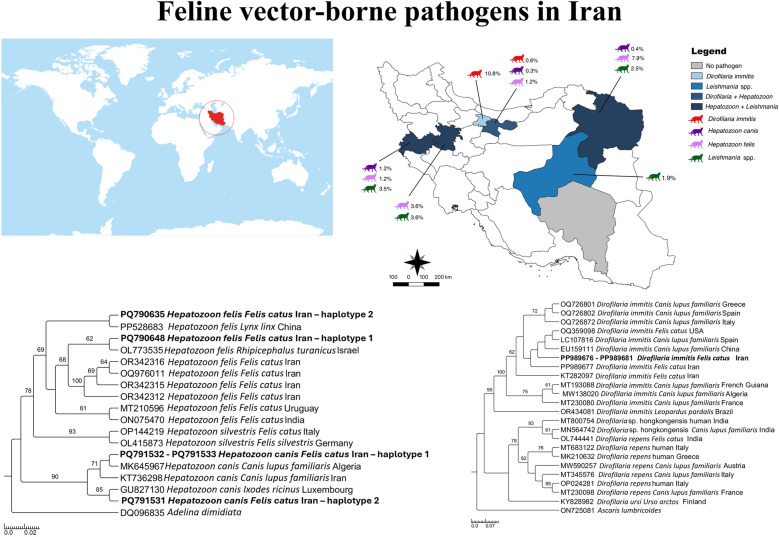

## Background

Vector-borne pathogens (VBPs) are of growing concern globally, not only due to the increasing incidence of the infections they may cause, but also because some of them have a zoonotic potential [1]. The growing trend of these infections is marked in Middle Eastern countries, not only because of the favorable ecological conditions for arthropods to thrive, but also because of the limited veterinary healthcare services [2–4]. In particular, several VBPs may infect cats by ticks (e.g., *Hepatozoon* spp., *Cytauxzoon* spp., *Babesia* spp.), mosquitoes (e.g., *Dirofilaria* spp.), and sand flies (e.g., *Leishmania* spp.) [1]. These pathogens are distributed in both Europe [5–7] and the Americas [8, 9]. For example, *Hepatozoon felis* is the main agent of feline hepatozoonosis worldwide [10], along with *Hepatozoon canis* [11], and to a lesser extent, *Hepatozoon silvestris*, the latter being described recently in Europe both in wild and domestic cats [6, 12, 13]. The infection by *H. felis* is usually asymptomatic, with exercise intolerance linked to skeletal muscle damage reported in some cases [14]. Conversely, several species of *Cytauxzoon* may infect domestic and wild felids worldwide [15–17], with *Cytauxzoon felis* circulating in sick cats from North America [18] and *Cytauxzoon europaeus*, *Cytauxzoon otrantorum*, and *Cytauxzoon banethi* in asymptomatic ones from Europe [16, 19]. As far as babesiosis, *Babesia felis* sensu stricto (s.s.), *Babesia leo*, and *Babesia galileei* (specifically infecting cats), as well as *Babesia canis* s.s., *Babesia gibsoni*, and *Babesia vogeli*, typical of dogs, have been diagnosed in cats [20–22], with different clinical presentations ranging from asymptomatic animals to clinical cases with lethargy, hemolytic anemia, and pyrexia [20].

While most of the abovementioned feline tick-borne pathogens circulate within the animal interface, pathogens transmitted by sand flies and mosquitoes tend to infect humans as well, raising concerns about the role played by cats in their epidemiology [7, 23]. In the case of *Leishmania infantum*, cats are considered secondary reservoir hosts [23–26] and also a source of infection for the sand fly vectors [27]. Besides the public health relevance, feline leishmaniosis represents a challenge for veterinarians, being characterized by unspecific pathological alterations and complex clinical diagnoses [28]. In addition, in Western Asia, cats may become infected with *Leishmania tropica,* and *Leishmania major*, thus playing a role as potential reservoirs of the infection [24, 29]. Cats can also become infected by the zoonotic *Dirofilaria immitis* and *Dirofilaria repens,* causing heartworm disease and subcutaneous dirofilariosis, respectively [30, 31]. For both *Leishmania* spp. and *Dirofilaria* spp., cats are considered not ideal hosts when compared with dogs, as they usually present low parasitic burdens [32, 33].

In Iran, a Western Asian country where VBPs are reported in dogs [4, 34, 35], data on the occurrence of feline vector-borne pathogens (FeVBPs) are limited to a few studies, mostly case reports, or performed in a small geographical area [36–39]. On that basis, this study was designed to overcome knowledge gaps on FeVBPs circulation in cats across the country.

## Methods

### Study areas and sample collection

**Fig. 1:**
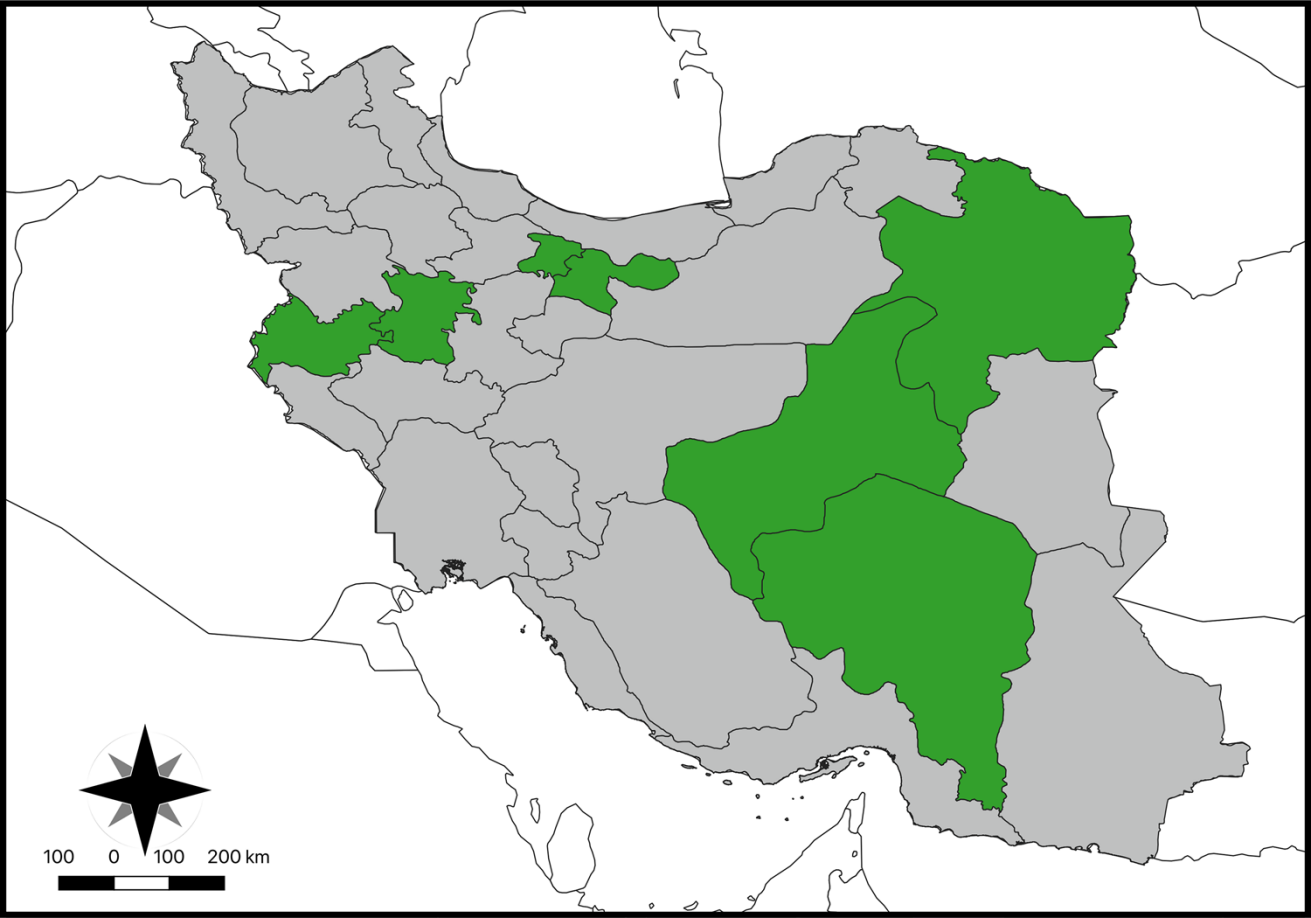
Geographical distribution of cats’ blood samples collected from seven provinces, namely Tehran, Khorasan Razavi, Kermanshah, Hamedan, Yazd, and Kerman (green) in Iran. The map was drawn using QGIS software version 3.26

From December 2018 to February 2023, a total of 848 blood samples were collected from cephalic or saphenous veins of cats with outdoor access, both client-owned (*n* = 511), and stray (*n* = 337) animals, coming from 7 provinces of Iran (Fig. 1). Blood samples were stored at –70 °C until DNA extraction. At the sampling, animal data were recorded in individual files, including sex, age, and housing condition. Animals were then grouped by age as kittens (up to 1 year, G1), young adults (from 1 to 6 years, G2), adults (from 7 to 10 years, G3), and seniors (> 10 years, G4).

### Molecular analyses

Genomic DNA was extracted from 200 μL EDTA-treated blood samples using the MBST DNA Kit (MBST, Tehran, Iran), following the manufacturer’s instructions. Samples were tested for piroplasms and *Hepatozoon* (*n* = 774) and filarioids (*n* = 848) by conventional polymerase chain reaction (cPCR) and for *Leishmania* spp. (*n* = 435) by real-time PCR (qPCR). All the details regarding primers, probes, and PCR protocols are reported in Table 1. Positive and negative controls were included for all the thermocycling reactions. The cPCR products were viewed by UV Imager (Transilluminator, Vilber Lourmat, France) after electrophoresis in a 1% agarose gel (SinaClon, Tehran, Iran) at 100 V for 60 min. Sequencing was run by Applied Biosystems 3500 Genetic Analyzer (Thermo Fisher Scientific, MA, USA) in Codon Genetics Laboratory (Tehran, Iran).

**Table 1:** Primers, target genes, and PCR conditions used in this study

Pathogen	Target gene	Primer (nucleotide sequence 5′-3′)	Method	Amplicon size (bp)	Reference
*Hepatozoon* spp.	18S rDNA	H14Hepa18SFw: GAAATAACAATACAAGGCAGTTAAAATGCT H14Hepa18SRv: GTGCTGAAGGAGTCGTTTATAAAGA	PCR	620	[40]
*Cytauxzoon* spp.	ITS2	*C. felis* F: TGAACGTATTAGACACACCACCT *C. felis* R: TCCTCCCGCTTCACTCGCCG	PCR	430	[41]
*Babesia* spp., *Hepatozoon* spp.	18S rDNA	Piroplasmid-F: CCAGCAGCCGCGGTAATTC Piroplasmid-R: CTTTCGCAGTAGTTYGTCTTTAACAAATCT	PCR	350–400	[42]
Piroplasmids	18S rDNA	GF2: GTCTTGTAATTGGAATGATGG GR2: CCAAAGACTTTGATTTCTCTC	PCR	560–610	[43]
*Leishmania* spp.	kDNA minicircle	LEISH-1: AACTTTTCTGGTCCTCCGGGTAG LEISH-2: ACCCCCAGTTTCCCGCC Probe: FAM-AAAAATGGGTGCAGAAAT	qPCR	120	[44]
Filarioids	*cox*1	NTF: TGATTGGTGGTTTTGGTAA NTR: ATAAGTACGAGTATCAATATC	PCR	648	[45]

### Phylogenetic analysis

For phylogenetic inference, sequences from the present study were aligned with those retrieved from GenBank using the MAFFT software version 7 [46]. The best evolutionary model was chosen under the Akaike information criterion (AIC) using the Cyber-Infrastructure for Phylogenetic Research (CIPRES) gateway (available at https://www.phylo.org/). Maximum likelihood phylogenetic analyses with 8000 bootstraps were performed using the iqTree gateway [47]. The phylogenetic tree edition and rooting (outgroup) were performed using TreeGraph 2.0 beta software [48].

### Statistical analysis

Prevalence analysis was performed using exact binomial 95% confidence intervals (CIs) for cPCR results. Possible associations between the VBP infection and risk factors, including city, sex, age, and living condition, were assessed by chi-squared tests using a free online tool (https://www.socscistatistics.com/tests/chisquare2/default2.aspx). *P* value < 0.05 was considered statistically significant.

## Results

**Fig. 2:**
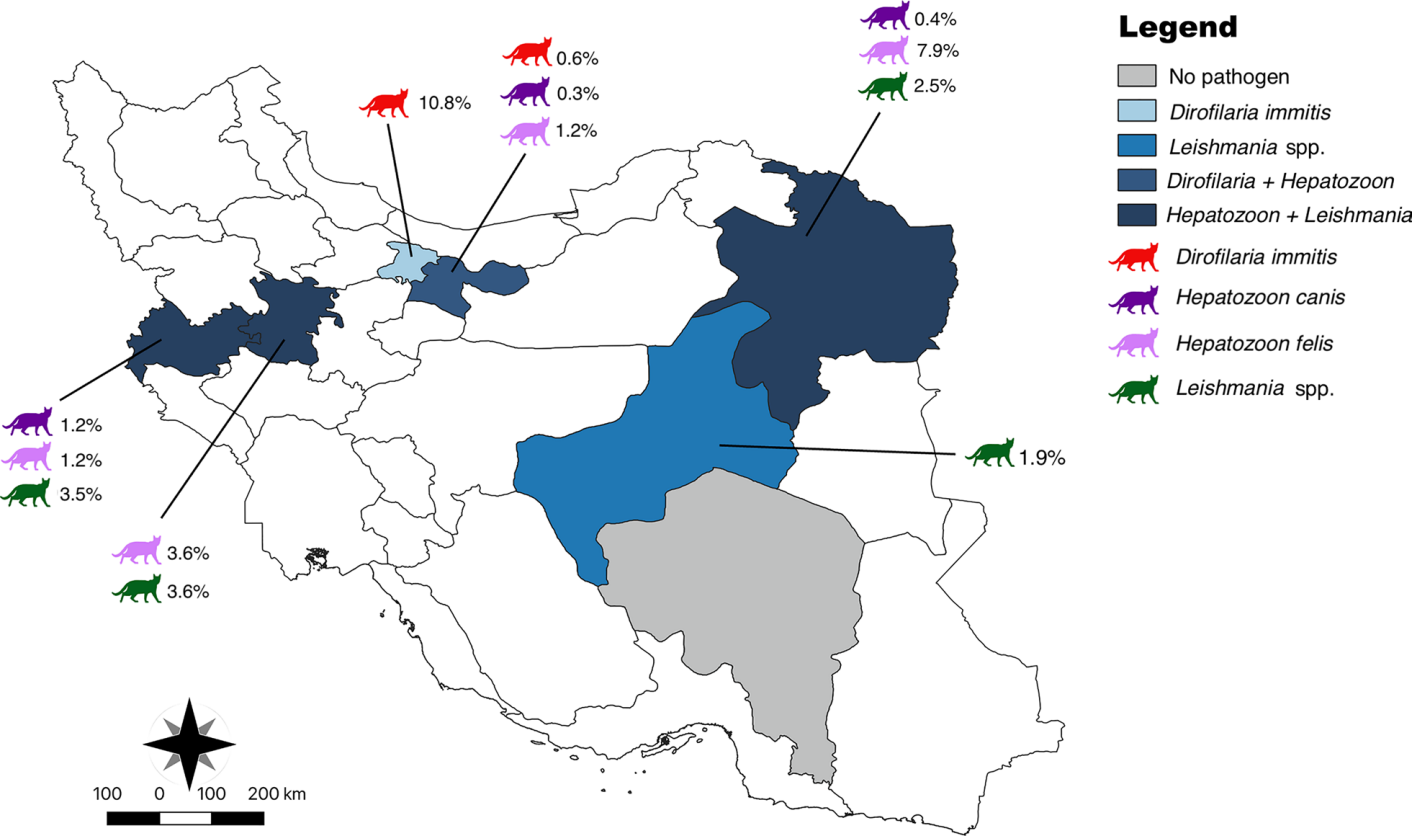
Distribution of cats infected with *Dirofilaria immitis*, *Hepatozoon canis, Hepatozoon felis*, and *Leishmania* spp. in Iran. The map was drawn using QGIS software version 3.26

**Fig. 3:**
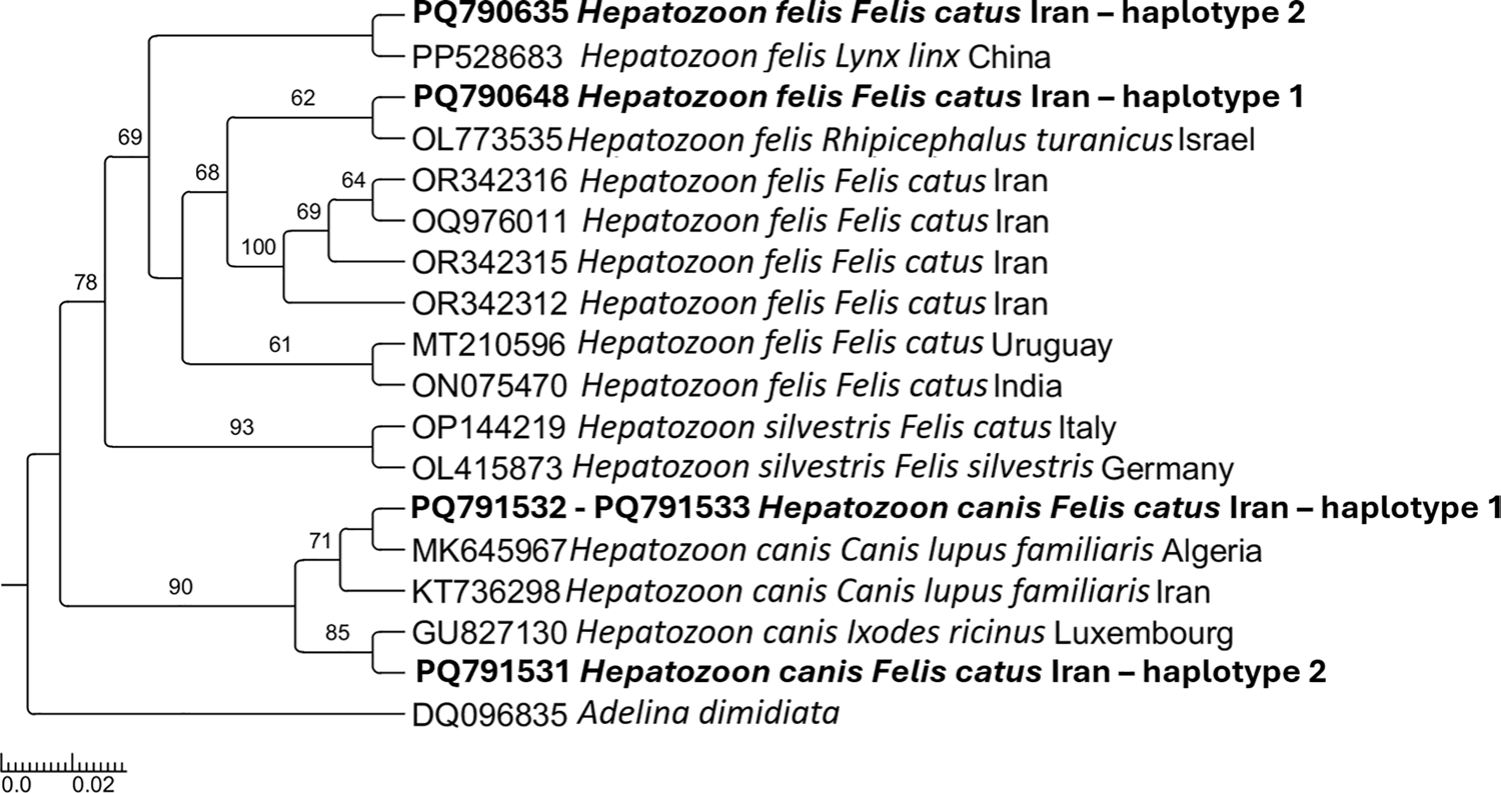
Phylogenetic trees inferred by maximum likelihood inference of the *Hepatozoon felis* and *Hepatozoon canis* sequences herein obtained by 18S rDNA gene. Sequences from the present study are marked in bold. *Adelina dimidiata* was used as an outgroup, and numbers at nodes indicate bootstraps values higher than 60

Overall, 5.3% of cats (i.e., 45/848, 95% CI 3.8–6.8%) scored positive for at least one VBP, with *Hepatozoon* spp. being the most common (3.7%, 29/774, 95% CI 2.4–5.1%), followed by *Leishmania* spp. (2.5%, 11/435, 95% CI 1.1–4%) and *D. immitis* (0.7%, 6/848, 95% CI 0.1–1.3%). Co-infection with *Hepatozoon* spp. and *Leishmania* spp. was recorded in one young adult female stray cat from Mashhad. *Hepatozoon* positivity was significantly higher in stray cats compared with owned ones (i.e., 4.2% versus 3%, *χ*^2^ = 15.31, *df* = 1,* P* = 0.00015). *Hepatozoon-*infected cats were found in the following provinces across the country: Khorasan Razavi in the east (20/239; 8.4%, 95% CI 4.9–11.9%), Hamedan in the west (2/55; 3.6%, 95% CI 0–8.6%), Kermanshah in the west (2/85; 2.4%, 95% CI 0–5.6%) and Tehran in the central-north (5/295; 1.7%, 95% CI 0.3–3.6%) of the country (Fig. 2). *Hepatozoon* positivity was significantly associated with geographical area (i.e., highest in Khorasan Razavi, *χ*^2^ = 14.3761, *df* = 3, *P* = 0.00157). Out of 29 *Hepatozoon* spp.-positive samples, 25 showed high identity with *H. felis*, three with *H. canis*, and one was low quality in sequencing and was not used in the phylogenetic analysis. The 25 sequences of 18S rDNA of *H. felis* were divided into two different sequence types, with the first (*n* = 16) showing > 99% of identity with *H. felis* detected in a wild felid from China (accession number [AN] PP528683) and the second (*n* = 9) showing 99% of identity with *H. felis* detected in a cat from Israel (AN KC138534). The two *H. felis* sequence types differed by three single nucleotide polymorphisms (SNPs) and the presence of three gaps (submitted to GenBank under accession numbers PQ790635 and PQ790648). The three sequences of *H. canis* were also divided into two different sequence types (submitted to GenBank under accession numbers PQ791531–PQ791533), with the first represented by two sequences showing 97% of identity with *H. canis* detected in a domestic dog from Algeria (AN MK645967) and the second represented by one sequence showing 96% of identity with *H. canis* detected in a questing female *Ixodes ricinus* tick in Luxembourg (AN GU827130). The *Hepatozoon* phylogenetic relationships are depicted in Fig. 3, with sequences of *H. felis* forming two different clades, with the first large clade composed of *H. felis* sequences from Israel and Iran and the second in a single clade with *H. felis* from China. The sequences of *H. canis* clustered with those from Algeria, Iran, and Luxembourg.

**Fig. 4:**
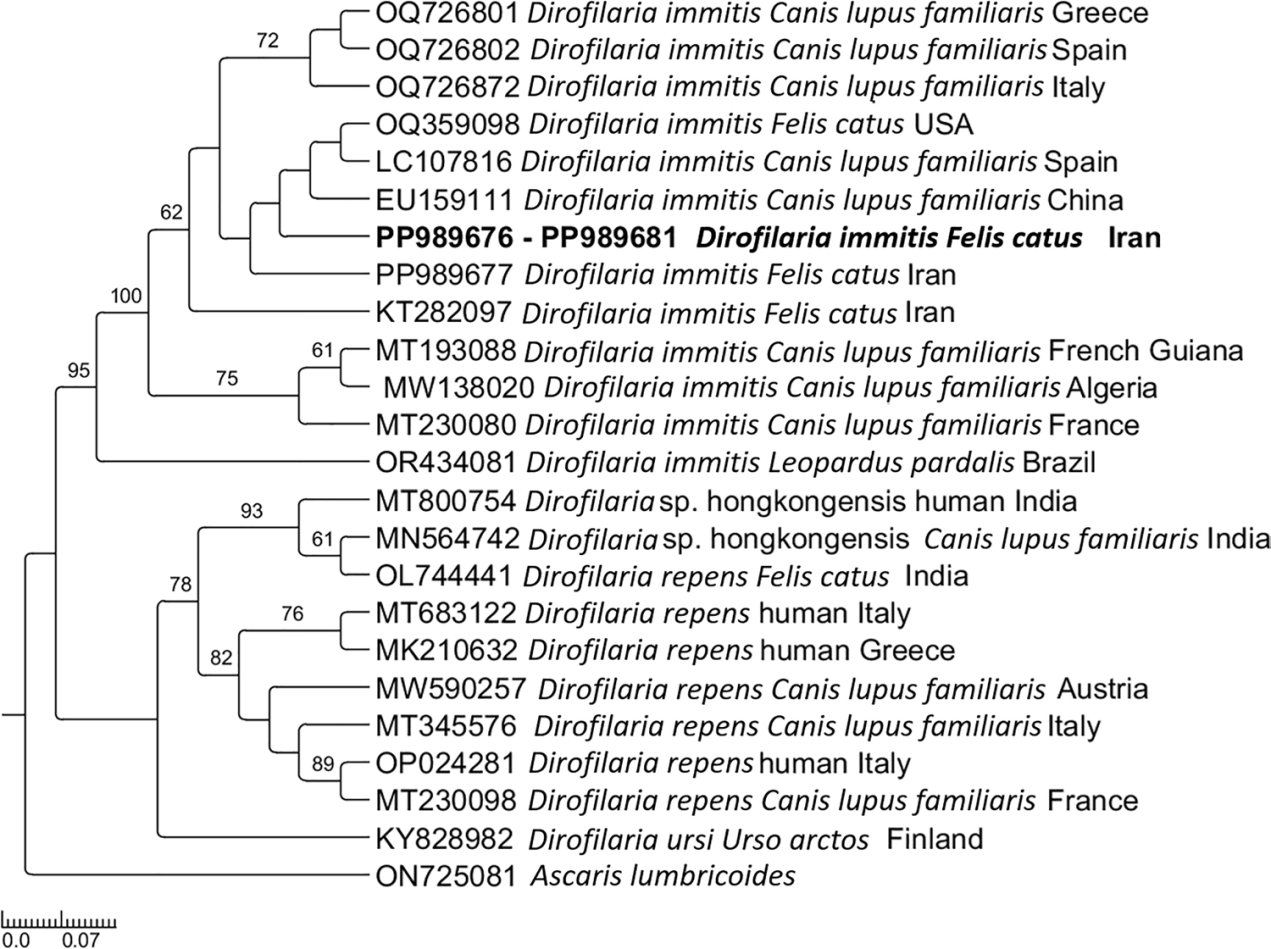
Phylogenetic trees inferred by maximum likelihood inference of the *Dirofilaria immitis* sequences herein obtained by *cox*1 gene. Sequences from the present study are highlighted in bold. *Ascaris lumbricoides* was used as an outgroup, and numbers at nodes indicate bootstraps values higher than 60

In total, six cats (0.7%; 95% CI 0.1–1.3%) from the provinces of Alborz (10.8%, 4/37, 95% CI 0.08–20.8%) and Tehran (0.6%, 2/332, 95% CI 0–1.4%) were positive for filarioids DNA (Fig. 2). Infected cats were stray, males, and young adults (1–6 years). All filarioid sequences showed 100% identity, with several sequences of *D. immitis* obtained from both cats and dogs worldwide (AN OQ359098, EU159111). The *Dirofilaria* spp. phylogenetic relationships are depicted in Fig. 4, with sequences of *D. immitis* (submitted to GenBank under accession numbers PP989676–PP989681), clustering in a large clade with *D. immitis* sequences obtained from different hosts worldwide.

DNA of *Leishmania* spp. was detected in the blood of 11 cats (2.5%, 11/435, 95% CI 1.1–4%), both stray (*n* = 6) and owned (*n* = 5), from Khorasan Razavi (2.6%, 6/227, 95% CI 0.6–4.7%), Kermanshah (3.6%, 3/84, 95% CI 0–7.5%), Hamedan (3.7%, 1/27, 95% CI 0–10.8%), and Yazd (2%, 1/49, 95% CI 0–6%) (Fig. 2). *Leishmania*-positivity was not statistically associated with housing condition (*χ*^2^ = 1.6204, *df* = 1,* P* = 0.203034), geographical location (*χ*^2^ = 0.3808, *df* = 3,* P* = 0.944174), age group (*χ*^2^ = 1.58, *df* = 2,* P* = 0.453839), or sex (*χ*^2^ = 0.5575, *df* = 1,* P* = 0.75673). All cats scored negative for *Cytauxzoon* spp.

## Discussion

The data presented provide an epidemiological picture of FeVBP circulation in cats, with *Hepatozoon* spp. being the most prevalent across the country, followed by *Leishmania* spp. and *D. immitis*. Phylogenetic analyses showed that most of the infected cats harbored *H. felis*, which can be related to the presence of hard ticks such as *Rhipicephalus sanguineus* sensu lato and *Rhipicephalus turanicus* across Iran [49, 50], both being suspected to be vectors of *H. felis* [51–53]. Accordingly, *Rh. sanguineus* s.l. are the most common ticks infesting domestic animals in Iran [54, 55]. The finding of *H. felis* infecting cats is consistent with previous reports from Iran [37] and other countries, such as Türkiye, Iraq, Greece, Portugal, Italy, and Germany [3, 6, 11, 52, 56]. In addition, we found cats infected with *H. canis*, as previously detected in both blood and ticks collected from dogs, in different regions of Iran [4, 34, 57, 58], which might be related to the genetic plasticity of *H. canis* [59]. The relatively low identity values observed (i.e., 96–97%) may suggest the circulation of a new genotype of *H. canis*. This hypothesis is supported by our phylogenetic analysis, in which the *H. canis* clades are robustly supported by high bootstrap values (i.e., > 70%), indicating a potential genetic divergence within *H. canis* detected in Iran. Moreover, hepatozoonosis was significantly associated with free-roaming lifestyle, supporting that stray cats may have an important role in the maintenance of the infection also in dog populations [6]. Circulation of the same pathogens in canine and feline populations in Iran stresses the importance of considering both animal species when planning prevention measures towards VBPs.

*Leishmania* spp. was the second most prevalent pathogen detected in cats, confirming its circulation, as suggested by former serological positivity retrieved from different areas of Iran (i.e., 14.3% in the northwest, 20.6% in the south, and 9.2% in the southwest) [39, 60–66]. Accordingly, a systematic review on leishmaniosis in Iran reported a higher rate of seroprevalence in domestic cats (19.3%) followed by dogs (12.5%), wolves (10.2%), foxes (9.9%), and jackals (6.4%) [67], suggesting a relevant role of cats in the epidemiology of the infection in the country. Nonetheless, serological cross-reactions could not be ruled out, as different species of *Leishmania* spp. (i.e., *L. infantum*, *Leishmania major*, and *Leishmania tropica*) are reported in Iran [68–70].

The molecular prevalence of *Leishmania* herein detected (i.e., 2.5%) is consistent with that recorded in cats from northwest Iran (i.e., 2%), but lower than in the south (i.e., 10%, 16.7%, 24.3%) [39, 63, 71, 72], possibly reflecting an uneven distribution of the competent vectors and the availability of reservoir hosts. Given the occurrence of common *Leishmania* species in both human and dog populations from Iran, as well as in the sand flies captured from different areas of the country [4, 70, 73, 74], data here obtained in cats further suggest the relevance of adopting preventive measures in animals to limit the parasite circulation in human premises. Hence, since pyrethroids are not available for preventing feline leishmaniosis, except for flumethrin-impregnated collars [75, 76], control strategies should be focused on reducing the populations of stray cats via spaying and neutering campaigns [77].

The low prevalence of *D. immitis* herein retrieved was expected, as cats are not the ideal host for this parasite [32, 78]. In Iran, the epidemiology of feline dirofilariosis is scarcely investigated, and to date, only four cats have been reported infected with *D. immitis* in East Azerbaijan [36], Khuzestan [79], and Ardabil [38]. On the contrary, canine and human dirofilarioses due to *D. immitis* and *D. repens* have been largely reported in the country [35]. However, the low prevalence herein detected might also be related to the low parasitic burden that cats usually harbor (i.e., often a single worm), leading to a lack of detectable genomic DNA at PCR [80]. Due to the abovementioned issues, a multitest diagnostic strategy, including serological tests, i.e., enzyme-linked immunosorbent assay (ELISA) and immunochromatography, is recommended for the diagnosis of feline dirofilariosis [81–84].

The absence of *Cytauxzoon*-positive cats can be related to the limited circulation of wild felid reservoirs, which are the main source of infection for the tick vectors [85, 86]. However, considering the increase in the synanthropic behavior of wild animals, the distribution and diversity of *Cytauxzoon* species in domestic and wild felids of Iran need to be monitored.

As a limitation of this study, we did not have data regarding possible infections of cats with feline immunodeficiency virus (FIV) and feline leukemia virus (FeLV), and in addition we could not obtain blood hematology and biochemistry tests and complete health checks to find the potential correlation between FeVBPs and clinical data. Additionally, the molecular method used in this study could not differentiate between *Leishmania* species. To identify the species circulating in cat populations in Iran, sequencing longer kDNA fragments or other markers, such as hsp70 or internal transcribed spacer (ITS), is recommended.

## Conclusions

To the best of our knowledge, this is the first large-scale epidemiological study on feline VBP infections in Iran, showing the circulation of *H*. *felis*, *H. canis*, *Leishmania* spp., and *D*. *immitis*. Overall, the data herein reported highlight the importance of performing ectoparasite control measures in owned cats, as well as the relevance of controlling feral animal populations by spaying and neutering campaigns.

## Data Availability

The datasets generated and analyzed during the current study are available in the NCBI—GenBank—Nucleotide platform (https://www.ncbi.nlm.nih.gov/genbank/) and can be accessed through the accession numbers provided in the article. Any additional data are available from the corresponding author (A.S.) on request.

## Abbreviations

FeVBP: Feline vector-borne pathogen
VBP: Vector-borne pathogen
EDTA: Ethylenediaminetetraacetic acid
cPCR: Conventional polymerase chain reaction
qPCR: Quantitative polymerase chain reaction
UV: Ultraviolet
DNA: Deoxyribonucleic acid
kDNA: Kinetoplast DNA
18S rDNA: Ribosomal DNA of the 18S subunit
ITS: Internal transcribed spacer
*cox*1: Cytochrome c oxidase subunit I
AIC: Akaike information criterion
MAFFT: Multiple alignment using fast Fourier transform
CIPRES: Cyber-Infrastructure for Phylogenetic Research
CI: Confidence interval
AN: Accession number
FIV: Feline immunodeficiency virus
FeLV: Feline leukemia virus
*df*: Degree of freedom
